# Development of decision support framework to prioritize GHG emission Scope in sub-national inventories

**DOI:** 10.1016/j.isci.2026.116002

**Published:** 2026-05-18

**Authors:** Muhammad Sumair, Muzaffar Ali, Tanzeel-ur Rashid, Guiqiang Li

**Affiliations:** 1 Department of Mechanical Engineering, University of Engineering & Technology, Taxila, Pakistan; 2 Department of Energy Engineering, University of Engineering & Technology, Taxila, Pakistan; 3 Department of Thermal Science and Energy Engineering, School of Engineering Science, USTC, Hefei, China

**Keywords:** environmental science, applied sciences, materials science

## Abstract

Greenhouse gas emission inventories are central to climate action planning. Existing accounting frameworks define emission Scopes but offer limited guidance on how to prioritize them for decision-making. This study develops a decision-support framework based on the analytic hierarchy process to evaluate and rank emission Scopes using stakeholder-informed criteria. Applied to Pakistan’s cement sector, the analysis identifies the feasibility of mitigation measures as the most influential criterion, and ranks Scope 1 emissions as the highest priority, followed by Scopes 2 and 3, respectively. Sensitivity analysis demonstrates that prioritization outcomes vary with changing emphasis on completeness, indicating that Scope selection is context-dependent, dynamic, and time-sensitive. The framework provides a transparent and adaptable approach to guide the development of balanced, policy-relevant emission inventories across sectors and regions.

## Highlights

• Development of a decision support framework for emissions Scope prioritization
• AHP applied to determine the contextual relevance of GHG emission Scopes
• Operationalizes the GHG Protocol relevance principle prior to inventory quantification
• Adaptable approach for sub-national and sectoral GHG inventory design

## Graphical abstract

**Figure:**
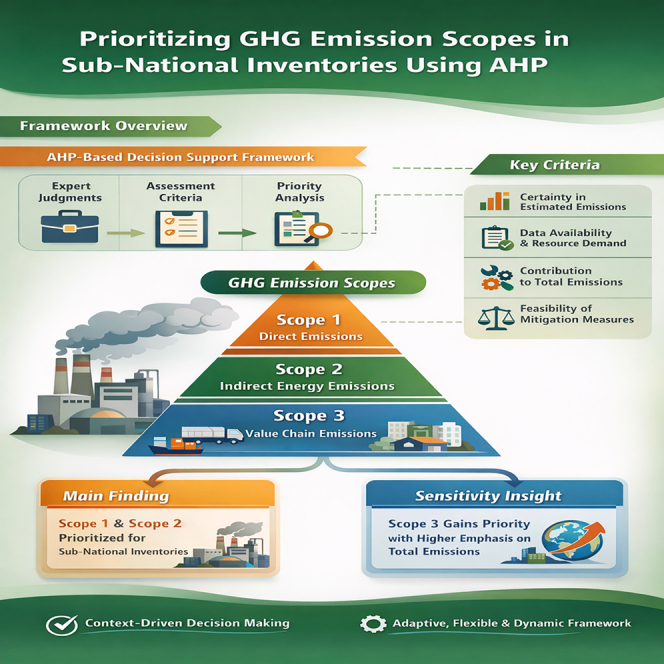

## Introduction

Articles 4 and 13 of the well-known Paris Agreement mandate the pledged nations to prepare and report Nationally Determined Contributions (NDCs) and greenhouse gas inventories.^1^ Although countries are mandated to develop national inventories, mitigation actions, and decarbonization roadmaps to achieve NDC targets rely heavily on sub-national inventories.^2^^,^^3^ Therefore, sub-national inventories including provincial, urban, and organizational scales are increasingly required^4^ to develop climate action plans. A well-designed emissions inventory quantifies emissions from relevant sources and activities within a defined geographic area and time frame.^5^^,^^6^^,^^7^ Accordingly, the inventories help identify emissions hotspots and set mitigation targets, making their quality and relevance central to effective climate policy.^8^^,^^9^

Persistent data gaps,^10^ limited institutional capacity, and resource constraints in developing countries often hinder the quantification of a detailed GHG emissions inventory.^11^^,^^12^ Addressing this challenge requires scientifically sound and contextually relevant approaches. This work proposes a relevance-based decision support framework to aid resource-constrained contexts in determining which emission Scope(s) is (are) most critical, enabling the quantification of fit-for-purpose sub-national inventories.

National inventories are typically required to achieve completeness by quantifying emissions from all the sources that come under a country’s jurisdictional boundary. It is a requirement of the United Nations Framework Convention on Climate Change (UNFCCC).^13^ However, the quantification of sub-national inventories becomes particularly challenging due to significant interactions of considered entities with others. These interactions may cause appreciable transboundary emissions (also called indirect emissions)^14^^,^^15^ from sources or activities that are not within the boundary or under the direct control of the reporting entity. Such complexity is further compounded by limited data availability, technical capacity, and financial constraints in developing countries.^11^

To clearly distinguish between direct and indirect emissions, the GHG Protocol Corporate Standard (referred to as the GHG Protocol henceforth)^16^ was the first to introduce the concept of emission Scopes at the sub-national scale. This framework divides emissions into three distinct Scope, defined later in discussion.

• **Scope 1:** The direct emissions from the sources owned or controlled by the company.
• **Scope 2:** This scope accounts for the indirect emissions associated with the purchase of off-site produced energy (steam and electricity).
• **Scope 3:** Scope 3 covers all other indirect supply chain emissions, including upstream and downstream emissions.

GHG Protocol introduces the principle *of relevance,* which is defined as “*Ensure that GHG inventory appropriately reflects the GHG emissions of the company and serves the decision-making needs of users.”*^16^ Principally, it suggests quantifying emissions from all such sources and activities that meaningfully impact the inventory objectives. However, existing protocols are often found emphasizing completeness over relevance. Also, they do not provide an explicit mechanism to determine the relevance of emission Scopes in the given context at a given point in time.

To examine this gap, a structured comparison of major GHG inventory protocols is presented in Table 1. The comparison assesses whether existing protocols: (1) apply the concept of emission Scopes, (2) provide guidance on Scope selection, and (3) include an explicit approach to determine Scope relevance before inventory development. Additionally, a review of the empirical sectoral and sub-national inventory studies is also presented in Table 2.

**Table 1:** Treatment of emission Scopes and approaches to Scope selection used for Scope selection across existing greenhouse gas inventory protocols

Protocol/Guideline	Primary Scale of Application	Use of Emission Scopes (1–3)	Guidance on Scope Selection	Explicit, Quantifiable Method for Determining Scope Relevance (Pre-Inventory)	Key Limitation in Resource-Constrained Contexts
IPCC Guidelines (1996, 2006, 2019)^17^^,^^18^^,^^19^	national/sub-national (territorial)	× does not use the Scope concept	N/A	N/A	Designed for completeness at the national scale; no mechanism to prioritize indirect emissions
GHG Protocol Corporate Standard (2001&2004)^16^	organizational/company	✓ explicitly defines Scopes 1–3	qualitative principle of “relevance”	× no operational or quantitative relevance-determination procedure	Scope inclusion left to practitioner judgment
GHG Protocol Scope 3 Standard (2011)^20^	organizational/company	✓ detailed Scope 3 categories	encourages the inclusion of Scope 3 where “relevant”	× no prioritization or screening framework	high data and coordination requirements
GHG Protocol Product Standard (2011)^21^	product	✓conceptually similar to Scope 3	N/A	N/A	data-intensive life cycle focus limits applicability
GHG Protocol Scope 2 Guidance (2015)^22^	organizational/company	✓detailed Scope 2 categories	encourages the inclusion of Scope 2 where “relevant”	× no decision-support method for relevance assessment	moderate data requirements
International Local Government GHG Emissions Analysis Protocol (IEAP, 2009)^23^	cities/communities/local governments	✓ uses Scopes 1–3	emphasizes completeness	× no relevance screening prior to inclusion	high data intensity for indirect emissions
International Standard for Determining Greenhouse Gas Emissions for Cities (2010)^24^	city/urban scale	✓ uses Scopes 1–3	emphasizes completeness	× no relevance-based selection mechanism	high data intensity for indirect emissions
Local Government Operations Protocol (2010)^25^	local governments	✓uses Scopes 1–3	emphasizes completeness	× no relevance screening	high data intensity for indirect emissions
Global Protocol for Community-Scale Greenhouse Gas Emission Inventories (GPC, 2014&2021)^26^	city/urban scale	✓ uses Scopes 1–3	emphasizes completeness	× no explicit relevance determination prior to reporting	resource-intensive indirect emission accounting
The Cement CO_2_ and Energy Protocol (2011)^27^	industry (cement plants)	✓ uses Scopes 1 and 2	recommends reporting where feasible	× no formal relevance-determination framework	Scope selection implicitly driven by data availability, although it explicitly acknowledges that Scope inclusion/exclusion is contextual sensitive
Cement Sector Emissions Calculation Tool (2005)^28^	industry (cement plants/companies)	✓ uses Scopes 1 and 2	recommends reporting Scopes where feasible	× no formal relevance determination	decision needs not explicitly considered
Cement Sector Scope 3 GHG Accounting and Reporting Guidance (2016)^29^	industry (cement plants)	✓ detailed Scope 3 categories	encourages inclusion where “relevant”	× no prioritization or screening framework	high data and coordination requirements

**Table 2:** Overview of selected sub-national GHG inventory studies applying emission Scopes, with emphasis on how Scope relevance is addressed

Sr. #	Author (Year), Location	Framework/Approach Adopted	Treatment of Emission Scopes	Key Observation Relevant to This Study
1	Wang et al. (2013),^30^ China	GHG Protocol Corporate Standard	quantified Scopes 1 & 2	Scope 3 omitted due to data limitations; no methodological justification or relevance-based screening provided.
2	Tangthieng (2017),^31^ Thailand	CSI Cement CO_2_ & Energy Protocol	quantified Scopes 1 & 2	Scope 3 emissions were not considered, and no rationale for exclusion was discussed beyond data availability.
3	Tan et al. (2022),^14^ China	CSI Cement CO_2_ & Energy Protocol	quantified Scopes 1 & 2	Scope 3 was excluded without a structured relevance assessment.
4	Wroclaw City Inventory (2013),^6^ Poland	IPCC + GPC	quantified Scopes 1–3	Inclusion of all Scopes; followed protocol completeness; no relevance-based prioritization discussed.
5	Beijing City Inventory (2012),^32^ China	GPC (dominant)	quantified Scopes 1 & 2; partially covered Scope 3	Several sectors omitted due to data gaps; decisions driven pragmatically rather than by a formal relevance framework.
6	Balaguera-Quintero et al. (2017),^33^ Chile	GPC (BASIC option)	Scopes 1 & 2 selected; partially covered Scope 3	Scope coverage determined primarily by data availability rather than decision-oriented relevance criteria.
7	Sanna et al. (2012),^34^ Italy	GPC (pilot version)	quantified Scopes 1–3	Full Scope coverage adopted, but no discussion on whether all Scopes were necessary for policy objectives.
8	Lu & Li (2012),^35^ China	IPCC Guidelines	quantified Scopes 1 & 2	Cross-boundary (Scope 3–type) emissions excluded; authors acknowledge resulting incompleteness but offer no selection rationale.
9	Bi et al. (2014),^36^ China	ICLEI Scope Framework	quantified Scopes 1 & 2	AFOLU and indirect emissions excluded due to complexity; relevance of exclusion not formally evaluated.
10	Li, Y et al.(2014)^37^	GPC	quantified Scopes 1–3	Authors explicitly recommend focusing on dominant sectors due to data constraints—implicitly acknowledging relevance—but without a formal decision framework.

Analysis of Tables 1 and 2 indicates that, although several protocols acknowledge the importance of relevance at a conceptual level, none translate this principle into a transparent or reproducible decision-support procedure. Also, Scope(s) selection in most empirical studies is either driven by the urge to ensure completeness or determined based on data availability, institutional capacity, or reporting convenience. It is also observed that some studies *implicitly* acknowledge the vitality of focusing on certain emission sources, and not others. However, none offer an *explicit* methodology for determining Scope relevance as a prerequisite to inventory development.

This observation leads to the following research question (RQ):

***RQ:*** *How can the contextual relevance of emission Scopes be determined systematically as a prerequisite for sub-national inventory development?*

To address this question, the present study develops a decision-support framework that operationalizes analytical hierarchy process (AHP), a well-established multi-criteria decision-making (MCDM) method. The proposed framework is not intended to quantify emission magnitudes or to replace existing inventory calculation methodologies. Instead, it addresses an *upstream* methodological gap by systematically prioritizing emission Scopes based on context-specific criteria. In doing so, the study responds to an underexplored issue in the literature: while the concept of Scope relevance is widely acknowledged in principle, its systematic determination prior to inventory development has not been *explicitly* operationalized. The framework is demonstrated through an application to Pakistan’s cement industry, an emission-intensive sector in a resource-constrained context. The case study illustrates how relevance-based Scope prioritization can support the development of “*balanced inventories,”* defined here as inventories that balance practical development challenges with climate action objectives.

The key contributions of this study are as follows.

• ***Problem identification:*** The study *explicitly* identifies the absence of a systematic mechanism for determining the relevance of emission Scopes prior to sub-national inventory development, a gap that has remained largely *implicit* in existing protocols and empirical studies.
• ***Conceptual reframing:*** This study reframes Scope selection as a decision-support problem rather than a choice driven solely by protocols or data availability. Accordingly, the study positions the determination of Scopes relevance as an *upstream* step in inventory design.
• ***Methodological integration:*** AHP is adapted and operationalized as a transparent and reproducible mechanism to translate qualitative stakeholder judgments and contextual constraints into prioritized emission Scopes.
• ***Dynamic applicability:*** The framework treats Scope prioritization as *non-static*, allowing reassessment as sectoral conditions, data availability, policy ambition, and mitigation maturity evolve over time.
• ***Practical relevance:*** Through application to Pakistan’s cement sector, the study demonstrates how relevance-based Scope prioritization can support the development of fit-for-purpose and balanced inventories in resource-constrained settings.

## Results

The decision support framework based on AHP was developed and implemented with the active participation of multiple stakeholders (explained in the method details section). Experts’ judgements, collected through well-designed questionnaires, were used to construct pairwise comparison matrices (PCMs) for both the evaluation criteria and the alternatives against each of the aforementioned four criteria. The results of the analysis leading to the determination of the relative weights of criteria and alternatives are presented later in discussion.

### Local and global priority vectors

**Figure 1:**
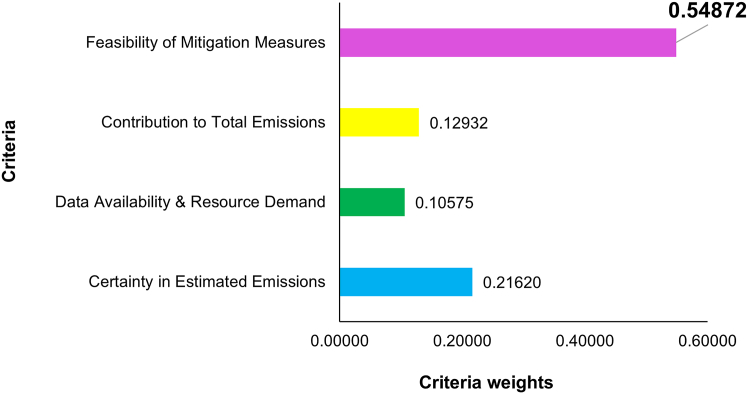
Aggregated local priority vectors of the four criteria used in the AHP framework to prioritize emission Scopes in Pakistan’s cement industry

**Figure 2:**
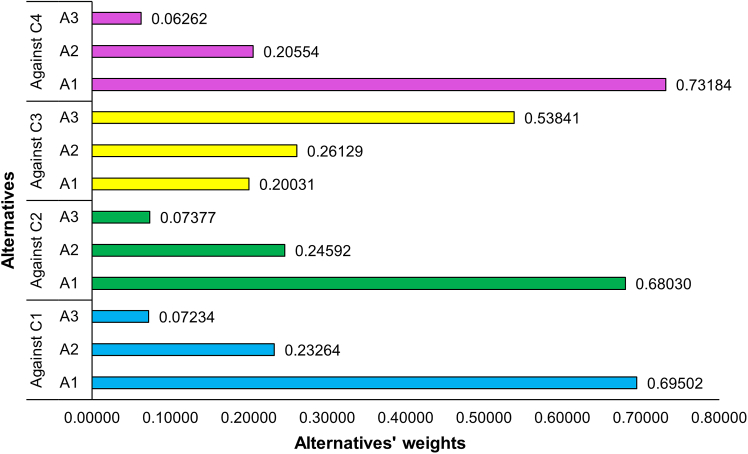
Aggregated local priority vectors of three alternatives—A1 (Scope 1), A2 (Scopes 1–2), and A3 (Scopes 1–3)—with respect to each of the four decision criteria

**Figure 3:**
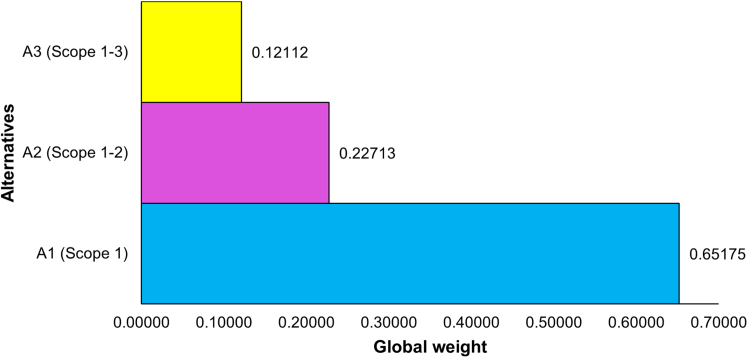
Aggregated global priority vector of the three alternatives–A1 (Scope 1 only), A2 (Scopes 1–2), and A3 (Scopes 1–3)– representing their overall priority for inclusion in the cement industry’s GHG emissions inventory

Of the fifteen responses received, four were identified as having an inconsistency with a consistency ratio greater than 0.1. These responses were returned to the respective respondents for careful revision. After resubmission, three responses remained inconsistent, which were excluded from further analysis. Consequently, the final analysis was based on twelve consistent responses. Local priority vectors for both criteria weights and alternatives were computed for each individual response (Table S5). The aggregated local priority vectors for criteria and alternatives are shown in Figures 1 and 2, respectively, while the global aggregated priority vector is illustrated in Figure 3. The consistency ratio was found to be 0.055 for the aggregated criteria matrix, and 0.054, 0.019, 0.029, and 0.057 for alternatives against each of the four criteria, respectively. As all CR values are below Saaty’s threshold of 0.1, the results are deemed consistent and acceptable.

Figure 1 illustrates that C4 (feasibility of mitigation measures) carries the highest weight (about 55%) among the four criteria. This shows that the inclusion or exclusion of specific emission Scopes in the emissions inventory is primarily driven by the fundamental purpose of inventory development-supporting the adoption of mitigation actions for effective climate action planning. Therefore, the Scopes with a higher probability of feasible mitigation measures must be prioritized in inventory preparation and must not be omitted under any circumstances. On the other hand, C2 (Data Availability & Resource Demand) has the lowest weight (about 11%), indicating that while data and resource constraints are important from a practical consideration viewpoint, they should not be used as the primary justification for the exclusion of any Scope. Instead, efforts must concentrate on generating or improving data for the relevant Scopes. Similarly, the weights of remaining criteria can also be interpreted accordingly to understand their influence on the decision-making process.

Figure 3 presents the overall ranking of alternatives, indicating that A1 (Scope 1) is the top-ranked alternative with a weight of 65.2% followed by A2 (Scopes 1–2) at 22.7% and A3 (Scopes 1–3) at 12.1%, respectively. These results indicate that, while considering both costs and benefits, prioritization of Scope 1 leads to the most balanced emissions inventory for the cement sector in Pakistan. The weight of A2 represents the incremental impact of including Scope 2 in addition to Scope 1, while the weight of A3 reflects the weight of Scope 3. Therefore, an emission inventory based on Scope 1 and 2 has the potential to account for more than 88% of the total representative value. Given its limited contribution and low feasibility for foreseen mitigation actions (as is illustrated in Figure 2), Scope 3 can reasonably be excluded from the cement sector’s emissions inventory in Pakistan without impacting the inventory’s representativeness and utility. While these results are context-specific, reflecting the dynamics of the Pakistani cement industry, the methodology can reasonably be applied in other countries to prioritize the emission Scopes according to their specific industry dynamics, regulatory frameworks, and mitigation objectives.

### Sensitivity Analysis

**Figure 4:**
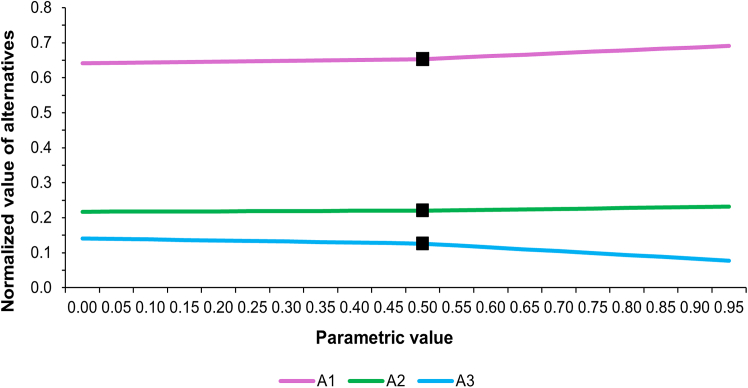
Sensitivity analysis shows the effect of varying the weight of C1 (certainty in estimated emissions) on the priority ranking of alternatives

**Figure 5:**
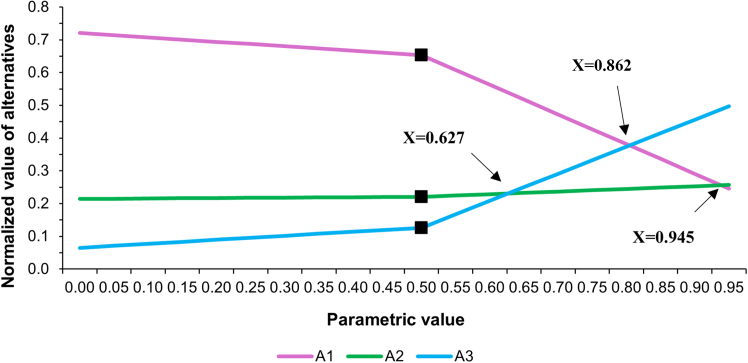
Sensitivity analysis shows the effect of varying the weight of C3 (contribution to total emissions) on the priority ranking of alternatives

Sensitivity analysis was conducted to evaluate the robustness of the analytical model with variation in the criteria weights. Dynamic sensitivity analysis was carried out using Super Decisions software to estimate how changes in criteria weights affect the priority ranking of alternatives. Figure 4 represents the sensitivity plot against C1 while Figure 5 illustrates the sensitivity plot against C3. As sensitivity plots of C2 and C4 reflect similar findings, those are included in Document S1 (Figures S1 and S2). In these plots, parametric values pertaining to the relative change in the criterion’s weight are plotted on X axis, while the Y axis shows the normalized priority values of alternatives. The baseline value of X = 0.5 represents the original criterion weight, by default. Shifting toward X = 1 or X = 0 reflects a relative increase or decrease in the criterion’s weight, respectively.

Sensitivity analysis shows a stable ranking of alternatives to changes in the weights of C1, C2, and C4. However, for Criterion C3 (Contribution to Total Emissions), there are several rank reversals, and its weight has a dominant effect on ranking. As the weight of Criterion C3 increases above X = 0.627 (which shows a 25.4% rise above its weight), its ranking line crosses A2’s ranking line. This implies that a 25.4% rise above its weight increases the priority ranking of A3 above A2. Further rising above its weight to a value of X = 0.805 finally makes it rank above A1 too and, thus, it finally becomes the highest-ranking alternative. This behavior does not undermine the framework’s conclusions but rather points out that Scope prioritization is conditional.

The rank reversals observed demonstrate a basic trade-off inherent in the design of an emissions inventory. Giving C3 greater weight reflects shifting the emphasis of the decision toward completeness of the inventory, consistent with the completeness principle of the GHG Protocol. Under these circumstances, a broader Scope coverage becomes naturally favorable, and A3, which covers all three Scopes, is ranked first. However, under baseline weighting—by expert judgment based on current data and institutional capacity and mitigation readiness—the feasibility and certainty considerations outweigh completeness, resulting in the prioritization of Scope 1–2 emissions.

In short, sensitivity results are not to be interpreted as contradicting the main findings. Rather, the interpretation of the sensitivity results is seen as evidence of the framework’s responsiveness to altering decision priorities. The analysis illustrates that the question of Scope prioritization is necessarily context-dependent based on the weights given to different criteria, such as feasibility vs. completeness. The sensitivity analysis thus strengthens the framework by explicitly stating the circumstances under which the alternative Scopes can become more favorable. It reinforces the framework’s function as a decision-support tool rather than a prescriptive ranking mechanism.

## Discussion

By developing and applying an AHP-based decision-support framework, this research provides a structured and transparent approach for prioritizing emission Scopes prior to inventory quantification. As the proposed AHP-based framework is not designed to quantify greenhouse gas emissions or to replace existing inventory calculation methods, the direct numerical validation against complete inventories is not applicable. Instead, it operates *upstream* of inventory development by providing a structured approach for prioritizing emission Scopes before quantification. This alignment with real-world inventory development practices provides an indirect empirical support for the framework’s practical relevance.

The empirical plausibility of the framework is done through the comparisons of the framework’s Scope prioritization outcomes with the reported patterns of Scope coverage in previous sub-national as well as sectoral emission inventories (Table 2). The previous works mostly quantify Scopes 1 and 2, while partially or completely excluding Scope 3, typically due to data and resource constraints and often without an explicit relevance-based justification. The framework’s results on the case study—Pakistani cement industry—are generally consistent with identified practices, and offer a valuable methodological improvement in that it makes Scope selection transparent and reproduceable.

Further contextual validation is obtained by comparing its results with the decarbonization roadmaps prepared for the global cement industry. Some of the most widely recognized roadmaps include “Technology Roadmap – Low-Carbon Transition in the Cement Industry”^38^ prepared by the International Energy Agency (IEA), and “The GCCA 2050 Cement and Concrete Industry Roadmap for Net Zero Concrete”^39^ prepared by the Global Cement and Concrete Association (GCCA). These roadmaps primarily emphasize the mitigation of Scope 1 and Scope 2 emissions, reflecting near- and mid-term feasibility under global-average conditions. In contrast, the European cement roadmap “Cementing the European Green Deal”^40^^,^^41^ prepared by CEMBUREAU—The European Cement Association, is increasingly shifting its focus to include Value Chain (Scope 3) emissions, consistent with the region’s advanced regulatory maturity, higher data availability, and progress in reducing direct emissions. This staged prioritization mirrors the framework’s results, which identify Scope 1–2 as immediate priorities for Pakistan’s cement sector, while recognizing Scope 3 as a delayed, longer-term consideration.

Applying the framework to the cement industry in Pakistan illustrates how using expert judgment alongside a formal multi-criteria approach can make informed Scope prioritization in a systematic and justified manner. The weight given to mitigation feasibility illustrates a key lesson for climate governance: the emission inventories should function to support strategic climate action rather than striving for completeness with limited possible translation into mitigation benefit.

It is important to note that the outcomes of prioritization are shaped by a relatively small but highly specialized expert panel. Although aggregating individual priorities reduces the influence of any single perspective, the results necessarily reflect the professional backgrounds, sectoral experience, and, most importantly, the current technological and regulatory context of Pakistan’s cement sector. Accordingly, the resulting Scope priorities should not be interpreted as universal, static, and definitive prescriptions. Rather, they represent a context-specific and time-sensitive assessment that can be revisited with the evolution of sectoral conditions, data availability, or policy priorities. In this way, the framework is intended to support informed decision-making, rather than producing fixed or immutable rankings.

It is also important to note that the proposed framework does not reinterpret the relevance principle to justify the permanent exclusion of Scope 3 emissions from all sub-national emission inventories universally. Instead, it provides a structured mechanism to prioritize emission Scopes *conditionally* and *contextually* at a given *point in time*. Accordingly, the results found from the case study reflect *current* sectoral conditions in a resource-constrained context that are incorporated through experts’ subjective judgments. Therefore, the outcomes should not be interpreted as normative guidance for all sub-national inventories or for the cement sector (for Pakistan as well as globally).

Moreover, it is worth mentioning that the results should not give the impression that Scope 3 emissions are of secondary importance to the consideration of climate accountability. Indeed, this study explicitly expresses the recognition that supply chain emissions are and will continue to play a vital role in the process of decarbonization as a whole, especially within the context of evolved regulatory settings.

Since the framework explicitly treats Scope relevance as dynamic rather than static, it supports the idea of staged prioritization—aligned with staged global decarbonization trajectories—via an initial phase where Scope 1 and Scope 2 emissions receive the earliest attention, with the eventual increasing focus on Scope 3 emissions after the establishment of the base mitigation paradigm.

Therefore, the proposed framework should be understood not as a screening method for the exclusion of supply-chain emissions from climate accountability but rather as a support tool for sequencing emission Scopes under real-world constraints. Its merit rests on rendering transparent, revisable, and context-consistent Scope prioritization while keeping compatibility with long-term decarbonization and supply-chain accountability.

While this case study is specific to the Pakistani cement industry and utilizes expert input specific to the context, the framework is intended as a decision-support model rather than a prescriptive tool that could be applied universally. Its potential applicability beyond this case study lies in the underlying logic of explicitly structuring trade-offs among competing considerations when assessing the relevance of emission Scopes.

The criteria used in this study reflect considerations that are commonly encountered in emission inventory development across sectors and regions. However, their relative importance is inherently context-dependent and would need to be re-evaluated for each new application, taking into account locally appropriate expert input and institutional conditions.

Consequently, the framework does not imply that similar prioritization outcomes would automatically emerge elsewhere. In sectors or regions where mitigation options for direct emissions have largely been exhausted, greater emphasis may reasonably be placed on value-chain emissions, which could result in different Scope prioritization outcomes. In this sense, the generalizability of the framework should be understood as procedural and methodological, rather than empirical or outcome-based. Applying it to other sectors or countries would therefore require contextual recalibration and validation, rather than the direct adoption of the results presented here.

This study advances the understanding of sub-national greenhouse gas inventory design and is based on two key premises: First, the quantification of emissions from all three Scopes is not always necessary for the development of sub-national GHG emissions inventories. Second, the relevance of emission Scopes should be determined contextually based on decision-making needs and mitigation priorities that may be time-sensitive. In broader terms, the framework contributes to ongoing debates revolving around the inventory by operationalizing the principle of relevance. It provides a link between the high-level protocol guide and the on-the-ground implementation process, especially in resource-constrained contexts. The framework does not supersede the existing inventory methodologies; the framework complements them by indicating which Scopes deserve priority attention before detailed quantification begins.

Finally, the framework is treated as inherently dynamic because the relevance of emission Scopes will continue to be reassessed with time due to increasing mitigation capacity, improving data availability, and stringent policy objectives. In this way, the proposed approach supports adaptive, context-sensitive climate governance, enabling sub-national actors to develop balanced inventories that align technical feasibility with long-term climate action objectives.

### Limitations of the study

This study has several limitations that should be acknowledged. The prioritization results are based on a relatively small but specialized expert panel, and although aggregation reduces individual bias, the outcomes reflect the perspectives and experience of the selected participants. The framework also relies on subjective judgments inherent to the analytic hierarchy process, which may introduce variability despite the application of consistency checks and sensitivity analysis. In addition, the case study focuses on Pakistan’s cement sector, and while the framework is methodologically transferable, the resulting prioritization outcomes are context-dependent and may differ across sectors or regions with varying data availability, institutional capacity, and mitigation maturity. Finally, the framework operates upstream of inventory quantification and does not assess the numerical accuracy of emission estimates, but rather supports structured decision-making for Scope selection under practical constraints.

## Resource availability

### Lead contact

Requests for further information and resources should be directed to and will be fulfilled by the lead contact, Muhammad Sumair (muhammad.sumair@uettaxila.edu.pk).

### Materials availability

This study did not generate new unique reagents.

### Data and code availability

• This article collects, processes, analyzes, and interprets the expert data that can be obtained from the lead contact.
• This article does not report original code.
• Any additional information required to reanalyze the data reported in this paper is available from the lead contact upon request.

## Acknowledgments

The authors would like to thank the experts from academia, industry, and policymaking institutions who participated in the AHP survey and provided valuable insights for this study. No external funding was received for this research.

## Author contributions

M. S. conceptualization, methodology, software, data curation, and writing - original draft. M.A.: validation, formal analysis, resources, writing - review and editing, and supervision. T.R.: conceptualization, methodology, supervision, and project administration. G. L.: writing - review and editing, supervision, and resources.

## Declaration of interests

The authors declare no competing interests.

## Declaration of generative AI and AI-assisted technologies in the writing process

During the preparation of this manuscript, the authors used ChatGPT was used primarily to improve language usage (rectifying spelling and grammatical errors) and assist in framing the graphical abstract. These tools were used solely for editing and presentation purposes and did not contribute to the conceptualization, methodology, analysis, or interpretation of the research. All content has been reviewed and verified by the authors, who take full responsibility for the accuracy and integrity of the work.

## STAR★Methods

### Key resources table

**Table:** 

REAGENT or RESOURCE	SOURCE	IDENTIFIER
**Software and algorithms**		

Super Decision 3.2	https://www.superdecisions.com/downloads/index.php?section=win3_0_beta	–

### Experimental model and study participant details

Omitted as our study does not involve biological models.

### Method details

#### Definition of alternatives

Corresponding to three emission Scopes, three alternatives (A1–A3) were defined:

1. A1: Scope 1 only
2. A2: Scopes 1-2,
3. A3: Scopes 1-3

The objective is to identify and select the most contextually relevant alternative amongst three defined alternatives. The evaluation of alternatives is done against multiple criteria, which systematically incorporate the costs and benefits associated with each alternative. Such decisions typically depend upon multiple criteria and thus MCDM techniques could provide a reasonable solution to such decision-making problems. An important aspect of decision making theory is the arisen complexity when there is more than one criterion impacting the selection of an alternative out of the several available (at least two).^42^^,^^43^ Such problems fall under multiple (or multi) criteria decision making (MCDM) category.^44^^,^^45^

#### Rationale for using AHP

Development of an emissions inventory involves several challenges including limited data availability, poor accuracy, and resource constraints. Such challenges may force the developer to quantify emissions under Scope 1 only, excluding the higher Scopes. However, omitting higher Scopes for the sake of simplicity can compromise the true objective of inventory development. Therefore, the proposed decision-support framework serves as a prerequisite for the development of a balanced inventory, in which Scope selection is aligned with available resources and context-specific objectives.

A closer examination indicates that determining the relevance of emission Scopes is inherently subjective, as it relies on expert judgment, experience, and informed intuition. Given that the intuition is very reliable if knowledgeable person provides judgement within an organized structure.^46^ Analytic Hierarchy Process (AHP) due to its robust structure that integrates the subjective input with an objective mathematical framework,^47^^,^^48^^,^^49^^,^^50^ is well suited for multi-criteria decision-making problems of subjective nature.^47^^,^^50^^,^^51^^,^^52^^,^^53^^,^^54^^,^^55^ This is especially true in fields like energy,^56^^,^^57^^,^^58^^,^^59^^,^^60^^,^^61^ sustainability,^62^ and environmental management.^63^^,^^64^^,^^65^ Such other applications can be found in.^66^^,^^67^^,^^68^^,^^69^^,^^70^^,^^71^^,^^72^ The feedback from multiple stakeholders–including policymakers, relevant ministries, industry experts and sectoral practitioners–were used for the assessment of relative importance of criteria and alternatives. Therefore, AHP was selected as the core decision support framework for emission Scopes’ prioritization in this study.

#### Structure of the AHP-based decision support framework

The hierarchical structure of AHP—based on simple mathematical principles—converts complex decision-making problems into simpler problems. The structure starts with defining a goal, for which a decision is to be made. On the next hierarchical level, properly defined criteria which are assumed to affect the decision are placed. These criteria are compared with each other pairwise following a quantitative framework. Based on the experts’ judgements—duly translated from subjective to objective nature—the pairwise comparison of various criteria is done to determine the weights of all criteria.

On the lowermost level of hierarchy, alternatives—the possible solutions of the problem— are placed.^73^ A pairwise comparison of alternatives is also done similar to the comparison of criteria. Such comparison of alternatives is made against each of the criterion, and an alternative having the highest score is typically selected as the optimum solution to the problem under consideration.^74^^,^^75^ A typical hierarchical structure followed in AHP is schematically shown in Figure S3.

#### Mathematical model of AHP

AHP is a subjective-cum-objective process, based on pair-wise comparison of various elements at each level of hierarchy through the following steps.

#### Criteria weights (W_i_) determination

A pair-wise comparison of criteria is conducted through establishing pair-wise comparison matrix (PCM). Supposedly, “m” number of criteria are defined for the given problem. Accordingly, a square pair-wise comparison matrix A of order m×m is formed as given in Equation 1. Each entry of the matrix A reflects the importance of one criterion relative to the other. For example, a_12_ shows the importance of criterion 1 relative to criterion 2 and so on. In general, a_ij_=k, where k is the numerical value according to Saaty scale (1–9), as given in Table S1. The PCM follows the reciprocity theorem i.e., the importance of 1^st^ criterion relative to the 2^nd^ is reciprocal to the importance of 2^nd^ relative to the 1^st^ criterion i.e. a_ji_=1/a_ij_=1/k. In this way, the matrix A is developed.

(Equation 1) $$A={\left[\begin{array}{cccc} 1 & a_{12} & \ldots & a_{1m}\\ a_{21} & 1 & \ldots & a_{2m}\\ \vdots & \vdots & 1 & \vdots \\ 1/a_{1m} & 1/a_{2m} & \ldots & 1 \end{array}\right]}_{m\times m}$$

Where a_11_=a_22_=a_33_=a_44_……a_mm_=, implying that each criterion is weighed against itself, thus the value should be 1.

The next step is to normalize the matrix A to obtain an eigenvector (normalized matrix) “N”, as given in Equation 2. The eigenvalues—the entries of the N matrix (b_ij_)—are obtained by dividing each entry of A matrix (a_ij_) by the sum of entries of the corresponding column in which a_ij_ lies^76^^,^^77^, as given in Equation 3.

(Equation 2) $$N={\left[\begin{array}{cccc} b_{11} & b_{12} & \ldots & b_{1m}\\ b_{21} & b_{22} & \ldots & b_{2m}\\ \vdots & \vdots & \vdots & \vdots \\ b_{m1} & b_{m2} & \ldots & b_{\text{mm}} \end{array}\right]}_{m\times m}$$

(Equation 3) $$b_{\text{ij}}=\frac{a_{\text{ij}}}{\sum_{i=1}^{m}a_{\text{ij}}}$$

Finally, a column matrix of weights (W)—called local priority vector,^78^ given in Equation 4—is developed by the row average of N matrix, as given in Equation 5, suggesting that the row average of first row of N matrix becomes W_11_ and so on. In this way, a column matrix of weights is developed.

Equation 4) $$W=\left[\begin{array}{c} W_{11}\\ W_{21}\\ \vdots \\ W_{m1} \end{array}\right]$$

(Equation 5) $$W_{i1}=\frac{\sum_{i=1}^{n}b_{\text{ij}}}{m}$$

Here W_11_ represents the weight of first criterion, W_21_ is the weight of second criterion and so on.

#### Evaluating alternatives against criteria

After the weight of each criterion is computed, evaluation of alternatives against each criterion one by one is the next logical step. Supposedly, “n” number of alternatives (A_1_, A_2_… A_n_) are compared pairwise against “m” number of criteria (C_1_, C_2_…. C_m_) one by one. The local priority vectors for alternatives are also obtained through same procedure as adopted for obtaining the corresponding vectors of criteria.

Based on the “n” alternatives, and “m” criteria, a n×m matrix is developed in such a way that first column of this matrix is populated by the product of the weights of “n” alternatives against first criterion (C_1_) and the weight of first criterion (C_1_) itself. Similarly, the second column is populated by multiplying the weights of all alternatives against 2^nd^ criterion (C_2_) with the weight of C_2_ itself and so on. Finally, a column matrix n×1—called global priority vector^77^^,^^79^^,^^80^—is formed by summing up all the elements in each row of previously formed n×m matrix. The highest value in this column matrix corresponds to the most prioritized alternative and so on. In this way, all alternatives are ranked and prioritized.

#### Determining the consistency ratio (CR)

Unlike many Multi-Criteria Decision-Making (MCDM) methods which inherently assume that the respondents are perfect in their responses, AHP acknowledges the probability of psychological error or discrepancies—technically called inconsistency—in individual responses. Therefore, consistency measurement is an important aspect of AHP.^81^ Thomas Saaty had introduced Consistency Index (CI) as a measure of consistency, given in Equation 6.

(Equation 6) $$\text{CI}=\frac{\lambda_{\max}-m}{m-1}$$

A perfectly consistent matrix must have CI=0. However, as the problem becomes complex, it becomes difficult to achieve consistency. Therefore, Saaty had suggested to compare the CI value for the problem under consideration as calculated from Equation 6 with the CI value expected from the matrix of the same order in question known as Random Index (RI) value, as given in Table S2. The ratio of original CI to the RI is termed Consistency Ratio (CR), given in Equation 7. According to Saaty, consistency is accepted if CR <0.1,^82^ otherwise, the comparison matrices must be re-developed and analysis must be repeated with another set of judgements.^83^

(Equation 7) $$\text{CR}=\frac{\text{CI}}{\text{RI}}$$

(Equation 8) $$\lambda_{\max}=\sum_{i=1}^{m}c_{\text{ij}}$$

The value of dominant eigenvalue (λ_max_) in Equation 6 is computed using Equation 8 i.e., by summing the elements of column matrix (AW). The column matrix AW is formed by multiplying the pair-wise comparison matrix A with column matrix of weights W, as shown in Equation 9. The order of column matrix would be m×1. Let the entries of this column matrix are c_11_, c_21_, c_31_….c_m1._

(Equation 9) $$\text{AW}=\left[\begin{array}{c} c_{11}\\ c_{21}\\ \vdots \\ c_{m1} \end{array}\right]$$

#### Design of hierarchical framework

As the assessment of alternatives (A1–A3) is based upon criteria, the formulation of these criteria is perhaps the most crucial aspect in the design of hierarchical framework for subjective problems. In most AHP applications found in the literature, the criteria are typically formulated based on relevant studies, while in some cases, they are defined through experts’ contributions.^84^ Further, it is considered good practice to get the criteria reviewed by the experts.^85^^,^^86^

#### Methodology for criteria formulation

In this work, the formulation of decision criteria followed a two-stage process combining a structured literature review with expert consultation. The objective was to identify a parsimonious set of criteria that collectively capture the key considerations influencing the relevance of emission Scopes in resource-constrained, sub-national inventory development.

In the first stage, an initial pool of candidate criteria was developed based on a comprehensive review of existing emission inventory studies, sectoral assessments, and relevant GHG accounting principles. This pool comprised approximately ten conceptual considerations commonly encountered in practice, as mentioned in Figure S4, and Table S3. In the second stage, the preliminary list of considerations, defined as criteria, was refined through structured expert consultation. The experts were asked to review the candidate criteria based on relevance, clarity, and potential overlap, if any. Consequently, several criteria were either merged or excluded to minimize redundancy.

#### Refinement and Consolidation of decision criteria

Those criteria that were considered redundant, largely overlapping, or weakly influential in the context of Pakistani cement industry were excluded from initial candidature criteria pool. As an example, Policy Relevance was initially assumed as an independent criterion. However, expert discussions showed that Policy Relevance could be expressed through the feasibility of implementing mitigation measures within the context of Pakistan’s cement sector. Accordingly, Policy Relevance was conceptually submerged within Feasibility of Mitigation Measures criteria. In a similar manner, criteria related to data accessibility, cost, and effort were consolidated into a single criterion capturing both data availability and associated resource requirements.

Through establishing consensus regarding the criteria applicability and interpretability, a set of four criteria was finalized to capture key determinants of Scope relevance in the given context. The initially proposed criteria, along with the rationale for their merging or exclusion, are documented in Table S3 and the complete methodology for criteria defining is presented in Figure S4.

The relevance of the final criteria is further supported by patterns observed in existing sectoral inventory studies. While the criteria themselves are broadly applicable across sub-national contexts, their relative importance is explicitly treated as context-dependent through stakeholder weighting.

A number of studies, such as those by Wang et al.^30^ (China), Tangthieng^31^ (Thailand),and Tan et al.^14^ (China) have worked on the development of emissions inventories for the cement industry. However, none of these studies have quantified all three Scopes; rather, due to unavailability of data, higher Scopes (Scope 2-3) have often been excluded. From this and other relevant studies, it appears that data availability is one key consideration and thus can be translated into criterion. However, the authors believe that only the unavailability of data cannot justify the exclusion of a Scope from the inventory. Instead, data should be arranged if a given Scope is relevant. Therefore, the resource requirement for data collection should also be considered an important factor. Hence, “Data Availability & Resource Demand” is defined as one criterion in this work.

Moving further, the authors emphasize that lower data availability and higher resource demand for data collection still do not justify the exclusion of a Scope, as long as the Scope is relevant and required in the local context. For example, if the cement sector emission inventory is developed with a particular focus on mitigating emissions associated with cement value chain (Scope 3), the unavailability of data for Scope 3 cannot justify its exclusion. Such an exclusion in this case will render the inventory redundant in serving its intended purpose. In short, the purpose of inventory development should be considered alongside. Therefore, following from above discussion, “Feasibility of Mitigation Measures” is defined as another criterion in this study.

The “Completeness” principle of GHG Protocol states that inventory should be as complete as possible, ensuring that no major emission sources are excluded provided they are relevant. Based on this principle, the criterion “Contribution to Total Emissions” has been defined to assess the significance of each Scope based on its share of total emissions. Similarly, following the principle of “Accuracy”, the criterion “Certainty in Estimated Emissions” has been introduced. This criterion evaluates the quality of developed inventory based on the certainty (or uncertainty) associated with emissions from each Scope. Below is given brief description of these criteria:

1. **Certainty in Estimated Emissions (C1):** Based upon the idea that the accuracy and certainty of estimated emissions inventory is crucial, this criterion is to investigate the effect of adding additional Scopes into emissions inventory on the certainty and accuracy of computed inventory. A Scope having higher certainty in its emissions data will receive higher score than the Scope(s) with greater uncertainty.
2. **Data Availability & Resource Demand (C2):** Acknowledging that data availability is an important factor to be considered while developing an emissions inventory, this criterion evaluates how easily data related to a particular Scope can be assessed or generated. It also considers the resources required-financial, human, and computational- for data collection and processing. A Scope with readily available data and lower resource demands will get higher scores.
3. **Contribution to Total Emissions (C3):** This criterion evaluates the relative significance of each emission Scope based upon its proportional contribution to total cement sector emissions. The Scopes having higher share to total cement sector emissions will score higher and thus have greater impact on overall inventory.
4. **Feasibility of Mitigation Measures (C4):** Knowing that the ultimate purpose of quantifying emissions inventory is to take mitigation measures, this criterion evaluates how feasible it is to implement mitigation measures for each Scope. Remember that cost-effectiveness, technological maturity, and regulatory support influence feasibility. The Scopes with more feasible and effective mitigation options will receive higher scores. The hierarchy of AHP for the current case study is shown in Figure S5.

#### Selection of experts

Once the hierarchical structure is designed through consensus, the next phase involves synthesizing or evaluating the framework to determine the priority ranking of alternatives. As implementation of AHP model relies on subjective input of experts to generate pairwise comparisons, selection of experts is critical. Keeping in view the expertise and diversity to ensure the credibility of the results, experts were drawn from three key domains:

1. Academia
2. Cement industry
3. Climate, environment, and sustainability policymaking

The shortlisting process was done very carefully according to the experts’ qualification, expertise, engagement with sector, and awareness of GHG accounting systems, including AHP. As far as the sample size is concerned, literature reveals that it is not required to survey a panel of large number of experts because surveying too many experts may make the synthesis procedure cumbersome and impractical.^87^ Many studies have selected expert panels of 8–12. For example,^87^ employed eight experts,^88^^,^^89^ used nine, and^90^^,^^91^ relied on twelve experts.

In this research work, twenty experts were short-listed to participate in the AHP study—eight experts from the academia and six experts each from the industry and the policy-making institutions. Of these, fifteen experts were willing to take part in the AHP Survey. After the consistency check, twelve consistent responses were selected to synthesize the results.

#### Handling of inconsistent responses

The consistency of each submitted response was evaluated, and if exceeded the acceptable limit, the response was returned to the respective respondent with a request to revisit their judgements. Theoretically, this exercise should continue until all responses achieve consistency. However, as repetitive responses submission can be impractical for experts, the authors opted to send responses back only once. Any responses which remained inconsistent after this step were excluded from further analysis. The responses fulfilling the consistency criteria were then processed to generate priority vectors in *Super Decisions* V3.2 Software.

Consistency filtering was done identically across all the participants and did not target any particular expert group. The final panel retained members from all three domains, ensuring the preservation of the intended multi-disciplinary balance. The individual experts may give more weight to criteria related to their professional roles, such as feasibility in the case of the industry experts, or mitigation relevance for the policy makers. Since the aggregated priorities reduce the effect of any single opinion, the results reflect a collective judgment in the context of the case study. The detailed composition of the expert panel before and after consistency screening is given in Table S4.

#### AHP survey and aggregation of judgements

Once the experts were selected and confirmed their willingness to participate in the AHP Survey, an AHP Survey Form was shared with them via email and Google Forms. The responses were then collected and processed for synthesis. Since multiple respondents were involved, individual judgements needed to be aggregated. The Aggregation of Individual Priorities (AIP) method, as given in Equation 10, was chosen for this purpose, as it is more suitable than Aggregation of Individual Judgements (AIJ) method when the group has diversity, and each respondent is assumed to respond independently.^92^^,^^93^

(Equation 10) wi=(∏k=1Nwik)1N

The methodology for complete solution and achieving the global priority vector leading to alternatives’ ranking is shown in Figure S6.

#### Sensitivity Analysis

Sensitivity Analysis (SA) is an important technique to evaluate the credibility of an analytical model by investigating the responsiveness of the output to variation in input. The subjective nature of input in AHP enhances the utilization of SA.^94^ By changing the priority weights of criteria, the effect on the priority ranking of the alternative is observed to determine the robustness of the framework.^95^^,^^96^^,^^97^ Once the local and global priority vectors were established, and priority ranking of alternatives was done, the dynamic sensitivity analysis was done in *Super Decisions* to identify the critical criteria, if any.

#### Practical Replication guide for non-expert users

This section provides a step-by-step implementation procedure to apply the proposed AHP-based framework intended for practitioners with limited experience in multi-criteria decision-making methods. Although *Super Decisions* software was used to facilitate computation in this study, the entire framework can be implemented using standard spreadsheet software such as MS Excel without requiring any specialized tools. Following is the sequence of steps to be followed:

1. Define the decision goal and alternatives (Scopes 1–3).
2. Finalize evaluation criteria through literature review and expert consultation.
3. Construct pairwise comparison matrices using Saaty’s 1–9 scale.
4. Normalize matrices and compute local priority vectors.
5. Check consistency (CR < 0.10); revise judgments if required.
6. Aggregate expert priorities using the geometric mean (AIP).
7. Compute global priorities and rank alternatives.
8. Perform sensitivity analysis to test robustness.

A fully solved numerical example, including the development of PCMs, normalization, consistency calculations, and aggregation steps, is provided in the Document S1: Methods S1: Step-by-step implementation of the AHP with a fully solved numerical example.

### Quantification and statistical analysis

The quantification, analysis and visualization of the results was carried out using a combination of Super Decisions and MSoft Excel. The details of the methods for aggregating data illustrated in figures and tables are presented in method details section, where appropriate.

## Supplemental information

Document S1. Figures S1–S6, Tables S1–S4, and Method S1

Table S5. Respondent-wise AHP local priority vectors, related to Figures 1–3
