# Supplementary material for: Development of decision support framework to prioritize GHG emission Scope in sub-national inventories
Source: iScience. 2026 May 18;29(6):116002. doi: 10.1016/j.isci.2026.116002 (PMC13197637; doi:10.1016/j.isci.2026.116002)
Supplement: Document S1. Figures S1–S6, Tables S1–S4, and Method S1 [file mmc1.pdf]

iScience, Volume *29*

Supplemental information

Development of decision support framework to prioritize GHG emission Scope in sub-national inventories

Muhammad Sumair, Muzaffar Ali, Tanzeel-ur Rashid, and Guiqiang Li

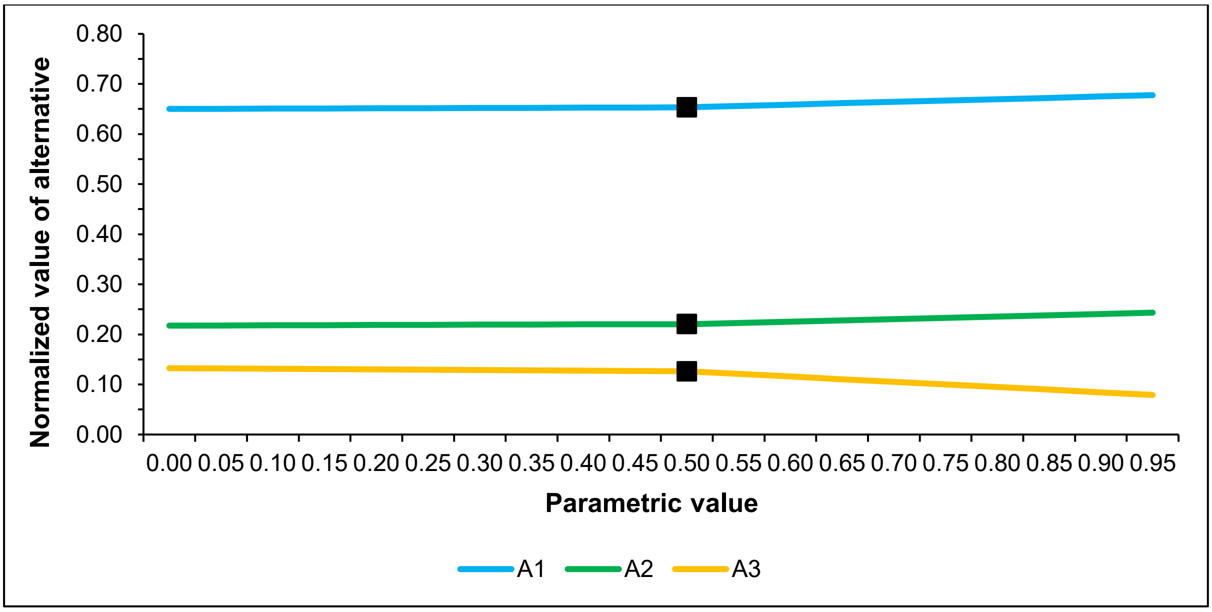

Figure S1. Sensitivity analysis with variation in weight of C2, related to Figures 4 and 5

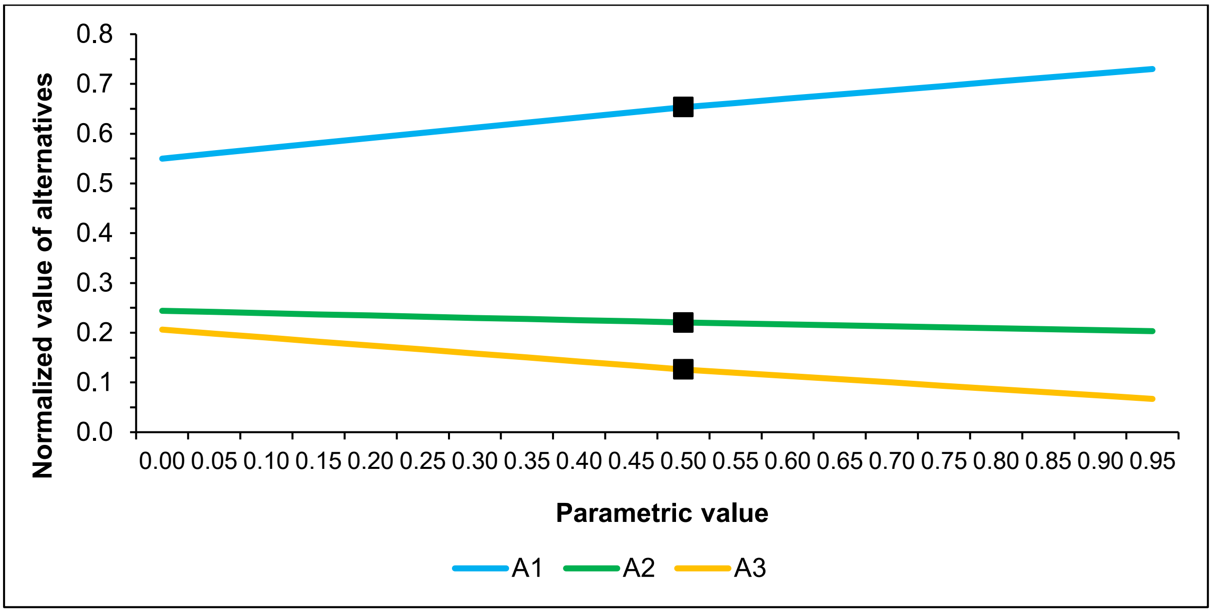

Figure S2. Sensitivity analysis with variation in weight of C4, related to Figures 4 and 5

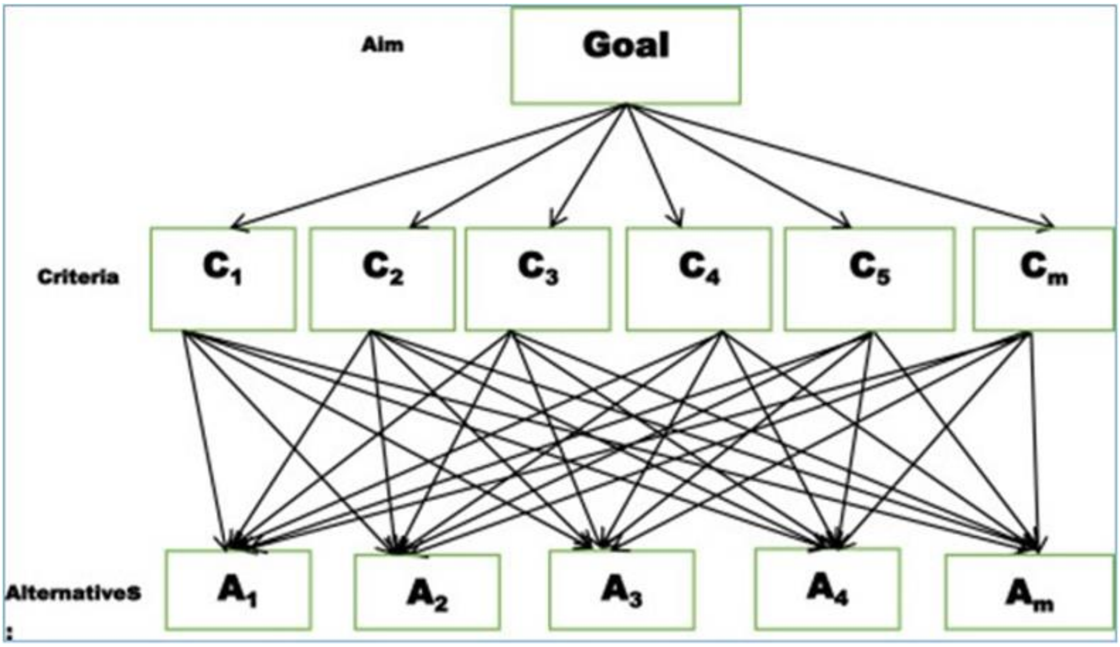

Figure S3. Typical Hierarchy of Analytic Hierarchy Process (AHP) [1], related to Star Method section

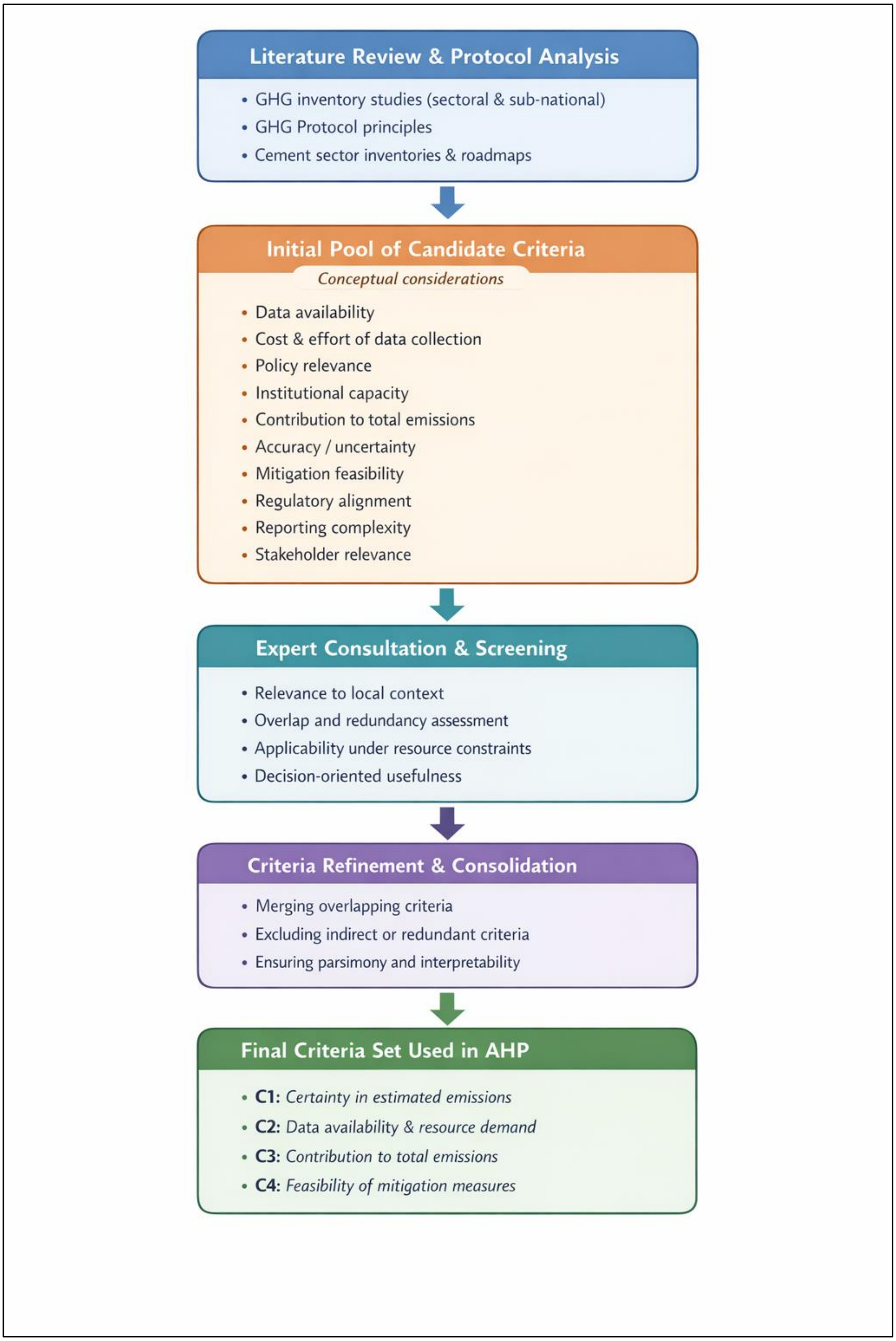

Figure S4. Schematic of criteria defining, refining and finalization used for AHP, related to Star Method section

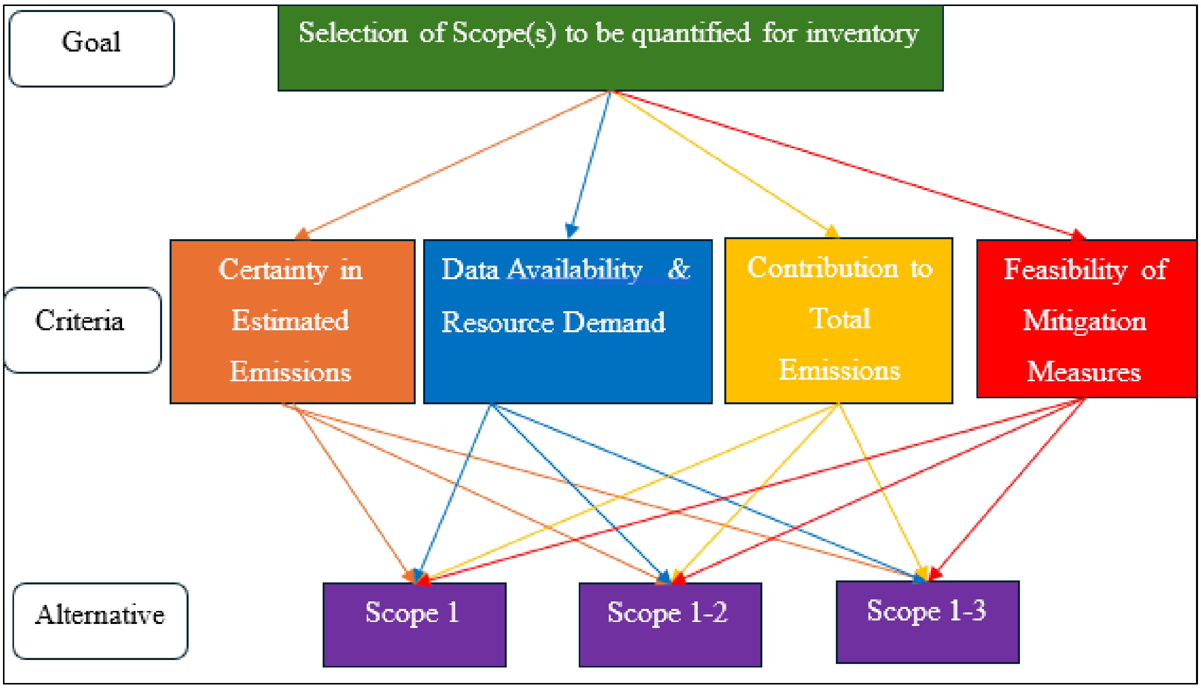

Figure S5. Hierarchical structure of the AHP-based decision framework to prioritize emission Scopes for the cement industry's GHG inventory, related to Star Method section

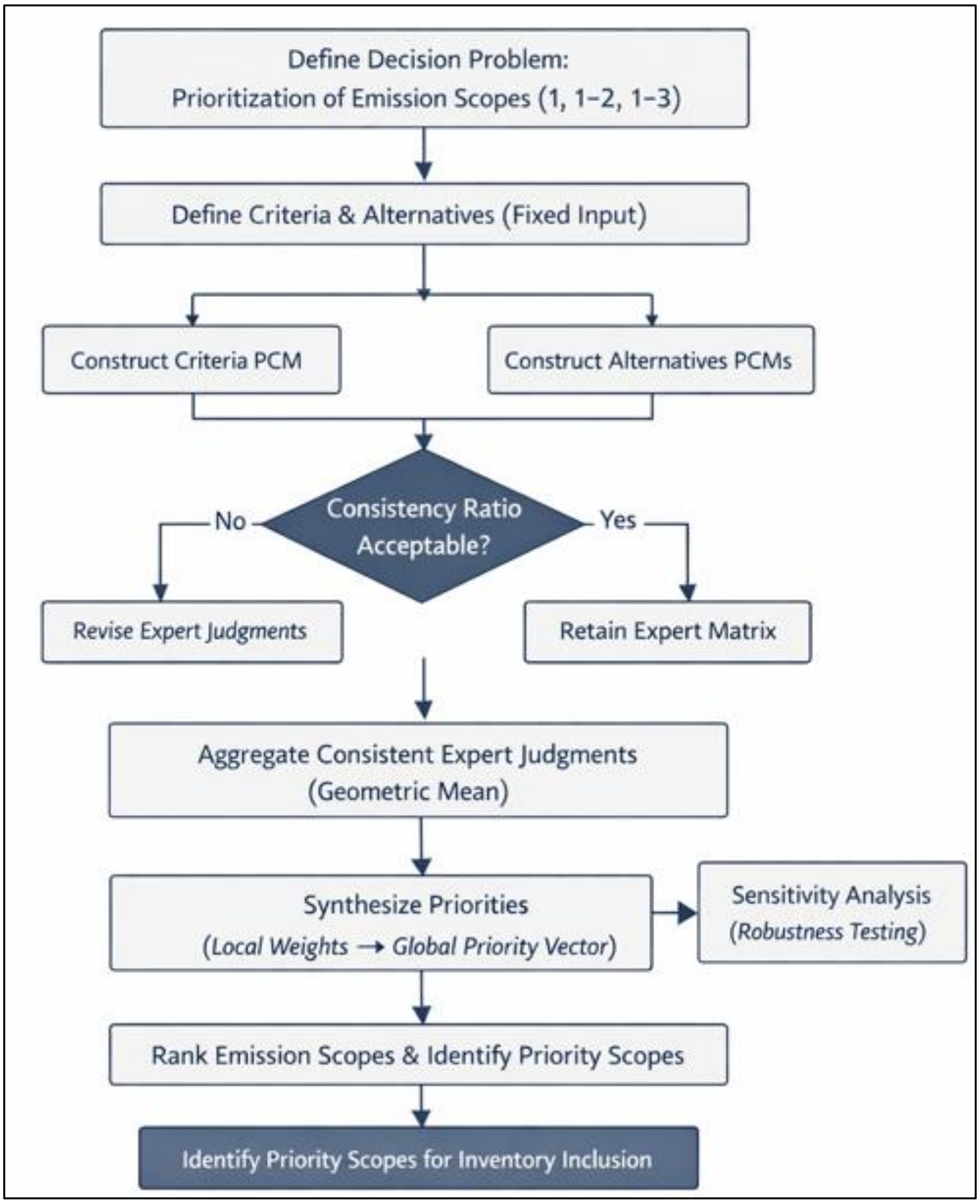

Figure S6. Flow diagram representing step-by-step implementation of the AHP-based decision support framework to prioritize emission Scopes

Table S1. Thomas Saaty Scale to transform the subjective preferences into numerical data [2], related to Star Method section

Scale	Inverse Value	Decimal Value	Description of Preferences
1	1/1	1.000	Equal Importance
2	1/2	0.500	Equally to Moderate
3	1/3	0.333	Moderate Importance
4	1/4	0.250	Moderately to Strong
5	1/5	0.200	Strong Importance
6	1/6	0.167	Strongly to Very Strong
7	1/7	0.143	Very Strong Importance
8	1/8	0.125	Very Strongly to Extreme
9	1/9	0.111	Extreme Importance

Table S2. Random Consistency Index (RI) of Saaty Scale[3-6], related to Star Method section

Matrix size	Random consistency index (RI)
1	0.00
2	0.00
3	0.58
4	0.90
5	1.12
6	1.24
7	1.32
8	1.41
9	1.45
10	1.49

Table S3. Initial criteria considered and rationale for refinement, related to Star Method section

**Initial Criterion**	**Source / Motivation**	**Final Treatment**	**Rationale for Merging or Exclusion**
Data Availability	Frequently cited in cement inventories	Retained (C2)	Core constraint affecting feasibility
Cost of Data Collection	Practical limitation	Merged into C2	Conceptually inseparable from data availability
Institutional Capacity	Developing-country constraint	Excluded	Reflected indirectly via feasibility and data access
Policy Relevance	Alignment with mitigation priorities	Merged into C4	Manifested through feasibility of mitigation
Mitigation Feasibility	Actionability of inventories	Retained (C4)	Central to decision-oriented relevance
Contribution to Emissions	GHG Protocol completeness principle	Retained (C3)	Captures materiality of Scopes

Initial Criterion	Source / Motivation	Final Treatment	Rationale for Merging or Exclusion
Accuracy / Uncertainty	GHG Protocol accuracy principle	Retained (C1)	Directly affects inventory reliability
Regulatory Alignment	Compliance considerations	Excluded	Overlaps with policy relevance
Data Transparency	Reporting quality	Excluded	Not determinant of Scope relevance
Stakeholder Pressure	Social expectations	Excluded	Context-specific and indirect

Table S4. Composition of Expert Panel after Consistency Screening, related to Star Method section

Expert Domain	Initially Shortlisted	Participated	Retained
Academia	8	6	4
Cement Industry	6	4	4
Policy & Sustainability	6	5	4
**Total**	20	15	12

Methods S1: Step-by-step implementation of the AHP with a fully solved numerical example

This section provides a fully solved numerical example to demonstrate the Analytical Hierarchy Process (AHP) procedure described in the main manuscript. The example uses hypothetical values and is intended solely for methodological illustration.

Goal: Selection of the most contextually relevant emission Scope

Alternatives

- A1: Scope 1 only
- A2: Scopes 1-2,
- A3: Scopes 1-3

Criteria

Assume three criteria (C1, C2, C3).

- C1 – Certainty in Estimated Emissions
- C2 – Data Availability & Resource Demand
- C3 – Contribution to Total Emissions

Step#1. Construction of Pairwise Comparison Matrix

Assume three criteria (C1, C2, C3). The pairwise comparison matrix A is constructed using Saaty's scale:

$$A = \begin{bmatrix} 1 & 3 & 0.5 \\ 0.33 & 1 & 0.2 \\ 2 & 5 & 1 \end{bmatrix}$$

Step#2. Normalization of the Pairwise Comparison Matrix

First, column sums are calculated:

$$\sum C1 = 3.33, \sum C2 = 9, \sum C3 = 1.7,$$

Each element of A is divided by its column sum to get normalization matrix (N)

$$N = \begin{bmatrix} 0.30 & 0.33 & 0.29 \\ 0.10 & 0.11 & 0.12 \\ 0.60 & 0.56 & 0.59 \end{bmatrix}$$

Step#3. Derivation of Local Priority Vector

The priority weights are obtained by averaging each row:

$$W = \begin{bmatrix} (0.30 + 0.33 + 0.29)/3 \\ (0.10 + 0.11 + 0.12)/3 \\ (0.60 + 0.56 + 0.59)/3 \end{bmatrix} = \begin{bmatrix} 0.30 \\ 0.11 \\ 0.58 \end{bmatrix}$$

Step#3. Consistency Evaluation

The weighted sum vector AW is computed:

$$AW = \begin{bmatrix} 1 & 3 & 0.5 \\ 0.33 & 1 & 0.2 \\ 2 & 5 & 1 \end{bmatrix} \begin{bmatrix} 0.30 \\ 0.11 \\ 0.58 \end{bmatrix} = \begin{bmatrix} 0.91 \\ 0.34 \\ 1.75 \end{bmatrix}$$

The consistency vector is obtained as:

$$\frac{AW}{W} = [3.03 \quad 3.09 \quad 3.02]$$

Thus,

$$\lambda_{max} = \frac{3.03 + 3.09 + 3.02}{3} = 3.05$$

The Consistency Index (CI) is:

$$CI = \frac{3.05 - 3}{3 - 1} = 0.025$$

For m=3, the Random Index (RI) is 0.58. Hence, the Consistency Ratio (CR) is:

$$CR = \frac{0.025}{0.58} = 0.043 < 0.1$$

Thus, the criteria judgements are consistent.

Step#4. Pairwise Comparison of Alternatives

Alternatives under Criterion C1 (Certainty in Emissions)

$$AC1 = \begin{bmatrix} 1 & 3 & 5 \\ 0.33 & 1 & 3 \\ 0.2 & 0.33 & 1 \end{bmatrix}$$

Local Priority Vector

$$W_{AC1} = \begin{bmatrix} 0.64 \\ 0.26 \\ 0.10 \end{bmatrix}$$

Alternatives under Criterion C2 (Data Availability & Resources)

$$AC2 = \begin{bmatrix} 1 & 2 & 4 \\ 0.5 & 1 & 3 \\ 0.25 & 0.33 & 1 \end{bmatrix}$$

Local Priority Vector

$$W_{AC2} = \begin{bmatrix} 0.57 \\ 0.29 \\ 0.14 \end{bmatrix}$$

Alternatives under Criterion C3 (Contribution to Emissions)

$$AC3 = \begin{bmatrix} 1 & 0.33 & 0.2 \\ 3 & 1 & 0.5 \\ 5 & 3 & 1 \end{bmatrix}$$

Local Priority Vector

$$W_{AC3} = \begin{bmatrix} 0.11 \\ 0.31 \\ 0.58 \end{bmatrix}$$

All alternative matrices satisfied the consistency requirement (CR < 0.10).

Step#5. Aggregation of Individual Priorities (AIP)

For group decision-making, priority vectors for both criteria and alternatives were aggregated using the Aggregation of Individual Priorities (AIP) approach:

$$w_i = \left(\prod_{k=1}^{N} w_i{}^k\right)^{1/N}$$

Aggregation was applied:

- once for criteria priorities
- separately for alternative priorities under each criterion

The aggregated vectors were normalized before synthesis.

Step#6. Global Priority Vector and Final Ranking

The global priority for each alternative was computed as:

$$P_i = \sum_{j=1}^{m} W_{c,j} \times W_{AC}$$

Substituting values gives

Alternative	C1 (0.30)	C2 (0.11)	C3 (0.59)	Global Priority
A1 (Scope 1)	0.192	0.063	0.065	0.32
A2 (Scopes 1–2)	0.078	0.032	0.183	0.29
A3 (Scopes 1–3)	0.030	0.015	0.342	0.39

Based on the global priority vector:

$$A_3 > A_1 > A_2$$

Therefore, under the hypothetical assumptions used in this example, Scopes 1–3 (A3) emerges as the most prioritized alternative. This numerical illustration demonstrates how different criteria weights can influence the prioritization of emission Scopes. The example highlights that when the contribution to total emissions (C3) dominates, higher Scopes may receive greater priority. This example is purely instructional and should not be interpreted as reflecting the empirical outcomes of the study.

Supplemental References

1. Afolayan, A.H., Ojokoh, B.A., and Adetunmbi, A.O. (2020). Performance analysis of fuzzy analytic hierarchy process multi-criteria decision support models for contractor selection. Scientific African *9*, e00471. https://doi.org/10.1016/j.sciaf.2020.e00471.

2. Alam, F., Azmat, M., Zarin, R., Ahmad, S., Raziq, A., Young, H.-W., Nguyen, K.-A., and Liou, Y.-A. (2022). Identification of Potential Natural Aquifer Recharge Sites in Islamabad, Pakistan, by Integrating GIS and RS Techniques. Remote Sensing *14*, 6051. 10.3390/rs14236051.

3. Badri, M. (2001). A combined AHP-GP model for quality control systems. International Journal of Production Economics *72*, 27-40. 10.1016/S0925-5273(00)00077-3.

4. Ammarapala, V., Chinda, T., Pongsayaporn, P., Ratanachot, W., Punthutaecha, K., and Janmonta, K. (2018). Cross-border shipment route selection utilizing analytic hierarchy process (AHP) method. Songklanakarin Journal of Science and Technology *40*. 10.14456/sjst-psu.2018.3.

5. Dodd, F.J., Donegan, H.A., and McMaster, T.B.M. (1993). A statistical approach to consistency in AHP. Mathematical and Computer Modelling *18*, 19-22. https://doi.org/10.1016/0895-7177(93)90123-G.

6. Gul, M., and Guneri, A.F. (2021). Hospital location selection: A systematic literature review on methodologies and applications. Mathematical Problems in Engineering *2021*, 1-14.
